# Single-Nucleus RNA Sequencing Reveals Muscle-Region-Specific Differences in Fibro-Adipogenic Progenitors Driving Intramuscular Fat Accumulation

**DOI:** 10.3390/metabo15040231

**Published:** 2025-03-28

**Authors:** Shuji Ueda, Chiaki Kitamura, Yuka Tateoka, Akinori Kanai, Yutaka Suzuki, Itsuko Fukuda, Yasuhito Shirai

**Affiliations:** 1 Graduate School of Agricultural Science, Kobe University, Kobe 657-8501, Japanitsuko@silver.kobe-u.ac.jp (I.F.); shirai@kobe-u.ac.jp (Y.S.); 2 Graduate School of Frontier Sciences, The University of Tokyo, Kashiwa 277-8562, Japan; akkanai@edu.k.u-tokyo.ac.jp (A.K.); ysuzuki@edu.k.u-tokyo.ac.jp (Y.S.)

**Keywords:** intramuscular fat, RNA sequencing, FAP, collagen, lipase, TGF-β, COL4A, ICAM1, TOX

## Abstract

Background: Ectopic fat deposition refers to lipid accumulation that affects metabolic function and tissue characteristics. Japanese Black cattle are distinguished by their high intramuscular fat content, which contributes to their distinctive character. However, the genetic mechanisms underlying these traits remain unclear. This study compared gene expression patterns in different muscle regions to identify genes associated with intramuscular fat accumulation. First, we conducted RNA sequencing to analyze differences in gene expression profiles among the sternocleidomastoid, pectoralis minor, and pectoralis major muscles. In addition, single-cell nuclear RNA sequencing was conducted to investigate the cellular composition of these muscle tissues. Results: Distinct gene expression patterns were observed among the different muscles. In the pectoralis, which contains a high proportion of intramuscular fat, adipocyte-related genes such as *FABP4*, *SCD*, and *ADIPOQ* were highly expressed. In addition, lipases such as *PNPLA2*, *LPL*, *MGLL*, and *LIPE* were predominantly expressed in intramuscular fat, whereas *PLA2G12A*, *PLD3*, and *ALOX15* were specifically expressed in myofibers. Moreover, a subclass of fibro–adipogenic progenitor cells that differentiate into intramuscular adipocytes was found to express genes related to microenvironment formation, including *ICAM1*, *TGFBRs*, and members of the *COL4A* family. Conclusions: This study provides novel insight into the genetic regulation of intramuscular fat accumulation. It improves our understanding of the molecular mechanisms underlying their distinctive meat characteristics.

## 1. Introduction

Ectopic fat deposition refers to fat accumulation in non-adipose tissues such as skeletal muscle, liver, pancreas, and heart. In humans, this phenomenon is associated with metabolic disorders such as insulin resistance, type 2 diabetes, and cardiovascular disease [1]. In livestock, ectopic fat deposition, including intramuscular fat (IMF), plays a crucial role in determining meat quality [2]. The relationship between intramuscular fat and meat quality has been studied in various livestock species, including cattle, goats, pigs, and chickens [3,4,5].

Japanese Black (Japanese Wagyu) beef is distinguished by its extensive marbling. This occurs due to the deposition of IMF via a distinct program of adipocyte accumulation in muscle tissues [6], a process that sets it apart from other breeds. Increased IMF deposition enhances meat tenderness and imparts a sweet and rich flavor, contributing to the distinctive taste of Japanese Black beef [7]. Accordingly, Japanese Black beef contains higher levels of free fatty acids, including oleic and linoleic acids, relative to other breeds [8]. These free fatty acids are thought to undergo peroxidation and hydroxylation reactions, which may contribute to the formation of aromatic esters such as lactones [9,10]. Lactones have been identified as key contributors to the sweet aroma of Japanese Black beef and play a crucial role in shaping its characteristic flavor [11].

IMF is a quantitative trait that is influenced by multiple genetic factors. Previous genome-wide association studies of Japanese Black cattle have identified several candidate genes associated with IMF, pinpointing a significant locus on chromosome 7 [12,13]. Beyond genetic factors, IMF formation is also affected by physiological factors such as gender, nutritional status, and hormone balance [14]. The analysis of mRNA, the transcriptional product of genes, is crucial for understanding the mechanisms underlying IMF formation. RNA sequencing (RNA-seq), a method for performing transcriptome analysis using next-generation sequencing (NGS), permits comprehensive examination of mRNA expression across all cells within a tissue [15]. RNA-seq also enables the simultaneous sequencing of thousands to millions of nucleotides, thereby facilitating the assessment of cellular state and the identification of key genes and enabling the inference of signal transduction pathways. In livestock production research, RNA-seq analysis has gained attention as a powerful tool for elucidating the molecular mechanisms underlying IMF formation and has been widely applied to various livestock species, including cattle [16], pigs [17], and chickens [18]. Recent advancements in single-cell RNA sequencing (scRNA-seq), which have enabled gene expression analysis at the single-cell level, have shifted the focus of IMF research from tissue-level to cellular-level investigations [19,20].

In recent years, extensive research has been conducted on the nutritional components and metabolites that influence meat flavor [21,22]. Alongside investigations into meat flavor, developing plant-based alternative meat products has gained attention due to increasing concerns regarding food security and sustainability [23]. While alternative meat products aim to replicate the flavor profile of conventional meat, they face challenges in reproducing its complex taste profile. Moreover, the sensory attributes of meat are determined by diverse tissue components, including variations in cell types and their spatial distribution [24,25]. The composition of muscle fibers and surrounding cells that constitute muscle tissue has been found to be significantly influenced by fibro–adipogenic progenitors (FAPs) [26]. FAPs are located around muscle fibers, possess mesenchymal properties, and exhibit multipotency, thus enabling their differentiation into intramuscular adipocytes [27]. Muscle tissue is also rich in extracellular matrix (ECM) components, particularly various collagens, whose expression is regulated in part by FAPs [28]. With a rapidly improving understanding of the relationship between muscle tissue and intramuscular fat, interest in IMF formation and its role in enhancing meat flavor and quality continues to grow [14,29].

This study aimed to analyze the mRNA expression profiles related to IMF formation in Japanese Black cattle using NGS technologies. These findings not only reveal specific gene expression patterns associated with IMF formation but also provide novel insights into the expression of lipid-metabolizing enzymes that contribute to the sweet aroma of Japanese Black beef.

## 2. Materials and Methods

### 2.1. Muscle Samples

Muscle tissue samples were collected from split carcasses of Japanese Black cattle within 30 min post-slaughter in a meat processing facility’s refrigerator. All samples were purchased with an average age of 32 months and a marbling score of 4 or higher, as evaluated by the Japanese Meat Grading Association standards. All cattle had access to drinking water and were fed a fattening diet containing corn, rice bran, and soybean meal. Additionally, all cattle were raised in barns designated for Japanese beef cattle production. Since the samples were obtained through commercial channels, this study did not involve animal breeding, dissection, or experimental procedures.

### 2.2. RNA Preparation from Muscle Tissue

Muscle samples were washed with Dulbecco’s phosphate-buffered saline (D-PBS; #049-29793, Fujifilm Wako, Osaka, Japan), after which subcutaneous fat and fascia were removed. For RNA extraction, muscle tissue was collected from four bulls and four cows, cut into approximately 10 mg (5 mm square blocks), and soaked in NAP buffer (19 mM EDTA, 18 mM trisodium citrate, 3.8 M ammonium sulfate).

Total RNA was purified from 100 mg of muscle tissue using the Maxwell RSC Simply RNA Tissue Kit (Promega K.K., Tokyo, Japan). RNA concentration was quantified using a NanoDrop spectrophotometer (Thermo Fisher Scientific K.K., Tokyo, Japan), and purity was assessed with a TapeStation (Agilent Technologies Japan, Tokyo, Japan). Samples with high RNA integrity (RIN) values were selected for RNA-seq analysis, fulfilling the criteria of total RNA ≥ 50 ng, A260/A280 and A260/A230 ≥ 1.6, and RIN ≥ 6.4 with a well-defined rRNA peak (Figure S1).

Purified RNA (10.0 ng) was amplified by PCR (7 cycles) using the Clontech SMART-Seq v4.0 Ultra Low Input RNA Kit (Takara Bio, Kusatsu, Japan) for the construction and sequencing of the total RNA-seq library. The amplified cDNA was purified using AMPure XP magnetic beads (Beckman Coulter, Tokyo, Japan). Finally, double-stranded cDNA (0.2 ng) was synthesized and barcoded using the Nextera XT DNA Library Prep Kit (Illumina K.K., Tokyo, Japan). The sequence library was validated using a fragment analyzer (Agilent Technologies).

### 2.3. RNA-Seq Analysis

Sequencing was performed on an Illumina NovaSeq 6000 platform using the NovaSeq 6000 S4 Reagent Kit v1.5 and the NovaSeq Xp 4-Lane Kit v1.5 (Illumina K.K.). Paired-end sequencing (150 bp read length) was conducted using NovaSeq Control Software (v1.7.5), Real-Time Analysis software (v3.4.4), and bcl2fastq2 conversion software (v2.20) from Illumina K.K. For each tissue type, samples from eight cattle were analyzed. To minimize potential biases, sequencing runs were conducted in a random order.

Sequence alignment and data analysis were performed using the Dragen Bio-IT Platform v3.7.5 (Illumina K.K.). The reference bovine genome (Bos taurus. ARS-UCD1.2.dna.toplevel.fa.gz; retrieved on 13 December 2023) and the corresponding gene annotation file (Bos taurus. ARS-UCD1.2.109.gtf.gz; retrieved on 15 December 2023) were used for alignment and analysis. These versions were selected to maintain consistency with previously reported RNA-seq data [15]. The mapping rate of read sequences to the reference genome exceeded 99.5% (Table 1). The sequencing quality, assessed by the percentage of bases with Q30 or higher, did not differ among muscle tissues (Figure S2).

Transcript per million (TPM) values between the two muscle tissues were statistically compared using t-tests to identify differentially expressed genes (DEGs). Statistical tests were applied to calculate *p*-values, and multiple testing correction was performed using the Benjamini–Hochberg method. Genes with q < 0.05 were defined as DEGs. Furthermore, genes that satisfied the criterion of |log_2_FC| ≥ 1 were selected to ensure biological significance.

Gene Ontology (GO) and Kyoto Encyclopedia of Genes and Genomes (KEGG) pathway analyses were performed on the DEGs using the Database for Annotation, Visualization, and Integrated Discovery (DAVID Knowledgebase v2024q2, https://davidbioinformatics.nih.gov/, accessed on 12 October 2024).

### 2.4. Single-Nucleus RNA Sequencing

Muscle tissue samples were washed in cooled D-PBS and dried with a paper towel. For nuclear extraction, muscle tissue was collected from a single cow, cut into approximately 10 mg (5 mm square blocks), transferred into cryovials, and rapidly frozen in liquid nitrogen. Nuclei extraction was performed using the Chromium Nuclei Isolation Kit (PN-1000494, 10× Genomics, Pleasanton, CA, USA). Muscle tissue was divid×d into five equal parts while cooling on dry ice. Each sample was placed in a sample dissociation tube containing 200 µL of lysis buffer from the kit and homogenized on ice with a pestle until evenly dispersed. An additional 300 µL of lysis buffer was added, followed by pipette mixing 10 times to ensure complete tissue dispersion. Nuclear extraction was then performed according to the manufacturer’s protocol.

The index sequencing library was prepared at the Life Science Data Research Center of the University of Tokyo using the Chromium GEM-X Single Cell 3′ Kit v4.0, Chromium GEM-X Single Cell 3′ Chip Kit v4.0, and the Dual Index Kit TT Set A (10× Genomics). The reference genome used for snRNA-seq was ARS×UCD2.0 (Bos taurus; GCA_002263795.4, https://www.ncbi.nlm.nih.gov/datasets/genome/GCF_002263795.3/ (accessed on 14 December 2024.). The sequence data were aligned using Cell Ranger (v9.0, update 18 November 2024). The analysis conditions for snRNA-seq are summarized in Table 2.

### 2.5. Bioinformatics Analysis

After sequencing and alignment, genomic data were analyzed using the R package Seurat (v4.0.4) (https://www.satijalab.org/seurat/ (accessed on 24 January 2025). Cells included for analysis were selected by filtering those containing between 1000 and 3750 genes per cell and a mitochondrial gene ratio of 2 or less. Gene expression data were then normalized to identify commonly expressed genes. In Seurat, gene expression data from the Round and Brisket were integrated, expression levels were normalized, and the data were scaled to minimize bias. Cells were clustered based on gene expression patterns. Clusters were visualized using principal component analysis with 2000 highly variable genes, followed by dimensionality reduction via uniform manifold approximation and projection (UMAP). Clusters were named by identifying cell populations based on the expression of well-known marker genes [19].

Single-cell differentiation trajectories were estimated using the Monocle 3 algorithm (https://cole-trapnell-lab.github.io/monocle3/ (accessed on 7 February 2025.). Following the tutorial, clustering and classification of single cells were performed, and changes in gene expression were analyzed along the pseudot ime trajectory [30].

### 2.6. Immunostaining Analysis

Tissues were lysed by sonication (15 s, four times) using an ultrasonic disruptor in RIPA buffer (10 mM Tris-HCl, pH 7.5; 1% NP-40; 0.1% SDS; 150 mM NaCl; 1 mM EDTA; 0.1% sodium deoxycholate) supplemented with a protease inhibitor (1 mM phenylmethanesulfonyl fluoride) and a phosphatase inhibitor cocktail (160-24371, Fujifilm Wako). To remove excess lipids, the lysate was mixed with 4.8 volumes of acetone, incubated at 4 °C with gentle inversion for 18 h, and subsequently centrifuged. Equal amounts of protein were subjected to SDS–polyacrylamide gel electrophoresis and transferred onto a PVDF membrane (Immobilon-P; pore size, 0.45 µm; Merck KK, Tokyo, Japan).

Polyclonal antibodies against TNNI2 (troponin I2, fast skeletal type; Accession No. A0AAA9RXA8) were produced by Eurofins Genomics Co. (Tokyo, Japan) using a peptide comprising residues 31 to 44 (ALPTRRAAPAKGHQ) as the immunogen. These antibodies were used in immunostaining experiments following IgG purification using a Protein A spin column (#APK-10A, Cosmo Bio Co., Tokyo, Japan). The specificity of the antibodies was assessed based on the position of the detected protein bands. Antibodies diluted 1:500 in Tris-buffered saline (150 mM NaCl, 50 mM Tris-HCl) containing 0.05% Tween 20 were incubated with PVDF membranes for 1 hr. The bound antibodies were detected by chemiluminescence using a secondary antibody conjugated to peroxidase (Jackson ImmunoResearch Laboratories, PA, USA) and ImmunoStar Zeta detection reagents (Fujifilm Wako) on the Limited-STAGE system (AMZ System Science, Osaka, Japan). Protein loading was confirmed by immunoblotting with a β-tubulin antibody, as previously described [31].

### 2.7. Tissue Staining

Paraffin-embedded sections (5 µm thick) were prepared from beef samples chemically fixed with a glyoxal solution (Falma, Tokyo, Japan). These sections were deparaffinized using a limonene-based solution (Fujifilm Wako). Hematoxylin and eosin staining was performed using Mayer’s hematoxylin solution and acid-extracted eosin alcohol solution (Fujifilm Wako).

## 3. Results

### 3.1. Comparison of Gene Expression in Different Muscle Tissues

**Figure 1:**
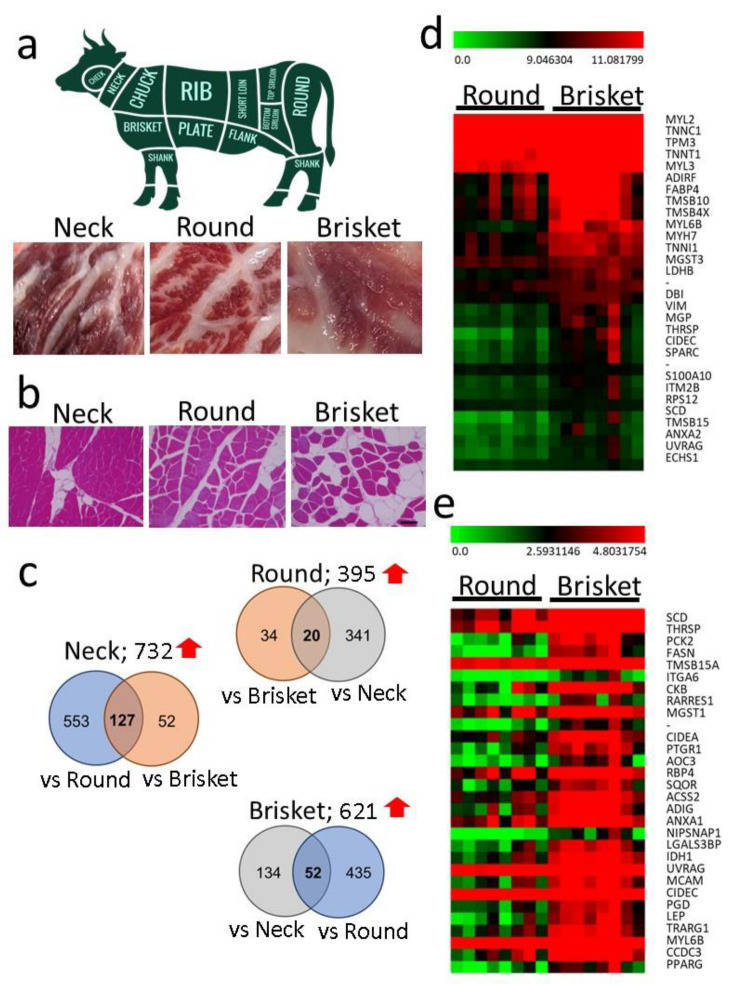
Comparison of gene expression using RNA-seq analysis. (**a**) Photograph showing samples of the sternocleidomastoid muscle (Neck), pectoralis muscle (Brisket), and adductor muscle (Round) of Japanese Black cattle. (**b**) Hematoxylin and eosin staining of paraffin-embedded sections. The scale bar represents 100 µm. (**c**) A Venn diagram illustrates the number of differentially expressed genes (DEGs) identified via pairwise comparisons among Neck, Round, and Brisket. (**d**) Heatmap illustrating gene expression differences between Round and Brisket. Heatmap of the top 30 highly expressed genes out of the 487 DEGs (vs. Round) in Brisket. (**e**) Heatmap of the 30 most specific genes with the highest expression levels among the 487 DEGs (vs. Round) in Brisket. Red indicates high expression, while green indicates low expression.

The sternocleidomastoid muscle (Neck), adductor muscle (Round), and pectoralis muscle (Brisket) were collected from the carcasses of slaughtered Japanese Black cattle (Figure 1a) and subjected to RNA-seq analysis. Hematoxylin and eosin staining revealed each tissue’s muscle fibers and IMF (Figure 1b).

RNA-seq analysis identified an average of 27,607 genes in muscle tissues. A total of 1041 (Neck vs. Round), 541 (Round vs. Brisket), and 365 (Brisket vs. Neck) DEGs were identified. The number of upregulated genes among the DEGs in the pairwise comparisons of Neck, Round, and Brisket is shown in the Venn diagram (Figure 1c). In Round, 54 of 395 DEGs were upregulated relative to Brisket, while in Brisket, 487 of 621 DEGs were upregulated relative to Round.

A heatmap was constructed to illustrate the top 30 DEGs identified in the comparison between Round and Brisket (Figure 1d). These highly expressed genes included *myosin (MYH)*, *troponin (TNN)*, and *tropomyosin (TPM)*, which encode proteins that constitute myofibrils. Another heatmap was generated to visualize the top 30 genes most highly expressed in Brisket relative to Round (Figure 1e).

**Figure 2:**
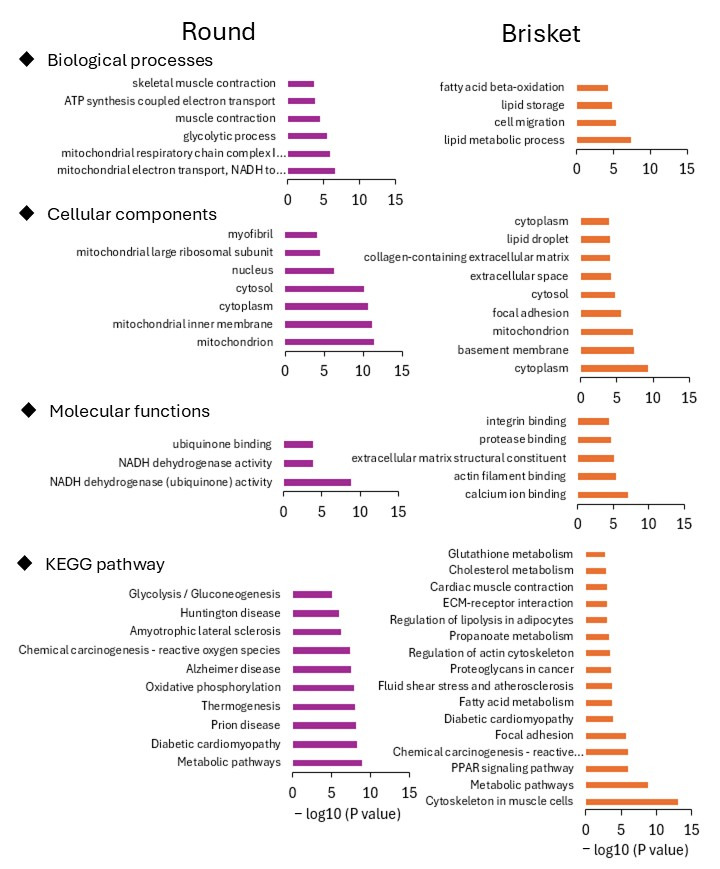
Annotation analysis of the adductor muscle and pectoralis muscle. Gene Ontology and KEGG pathway analyses were conducted using DAVID on upregulated genes in the adductor muscle (Round) and pectoralis muscle (Brisket).

Next, annotation and pathway analysis were conducted using GO and KEGG pathway data for upregulated genes in Round and Brisket (Figure 2). In the GO analysis of Round, terms such as “muscle contraction”, “mitochondrion”, and “myofibril” were identified, while the KEGG pathway category included “metabolic pathways related to protein synthesis”. In contrast, GO analysis of Brisket identified terms such as “lipid metabolic process”, “basement membrane”, and “lipid storage”, while the KEGG pathway category included “lipid metabolism pathways related to fatty acid metabolism” and “extracellular matrix-related pathways involving collagen”. GO analysis and KEGG pathway enrichment of genes with increased expression levels in the Neck are shown in Figure S3.

**Figure 3:**
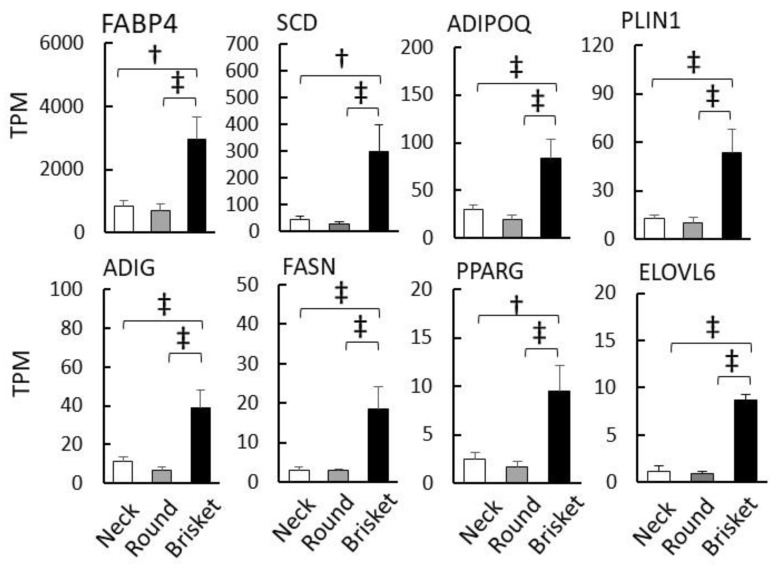
Upregulated genes in the pectoral muscle. Upregulated genes associated with lipid metabolism. The graph displays gene expression levels, represented as the mean TPM (transcripts per million). Error bars indicate ±SE. Significant differences are denoted as follows: ‡ *p* < 0.01, † *p* < 0.05 (Tukey’s HSD test, *n* = 8).

Annotation analysis identified multiple GO terms related to lipid metabolism in Brisket. Consequently, 52 genes were upregulated in Brisket compared to Neck and Round samples (Figure 1e), and are visualized in the heatmap shown in Figure S4. In Brisket, genes including *fatty-acid-binding protein 4* (*FABP4*), *adiponectin* (*ADIPOQ*), *perilipin 1* (PLIN1), and adipogenin (ADIG) exhibited significantly higher expression levels compared to Neck and Round samples (Figure 3). In addition, genes involved in lipid metabolism, such as *stearoyl-CoA desaturase* (*SCD*), *fatty acid synthase* (*FASN*), *very-long-chain fatty acid elongase 6* (*ELOVL6*), and *peroxisome proliferator-activated receptor gamma* (*PPARG*), were also significantly upregulated in Brisket.

### 3.2. Comparison of Cells Using Single-Nucleus RNA-Seq Analysis

Conventional scRNA-seq analyses are restricted by a microfluidic channel diameter of 40 µm, which limits the analysis of myofibers and mature adipocytes [32]. To address this limitation, we employed single-nucleus RNA sequencing (snRNA-seq) to investigate gene expression at the cellular level in the muscle tissues of Japanese Black cattle. Nuclei were extracted from Round and Brisket samples, enabling the analysis of nuclear gene expression. During data processing, dead cells and mitochondrial contamination were removed, yielding 32,771 nuclei from Round and 25,801 nuclei from Brisket.

**Figure 4:**
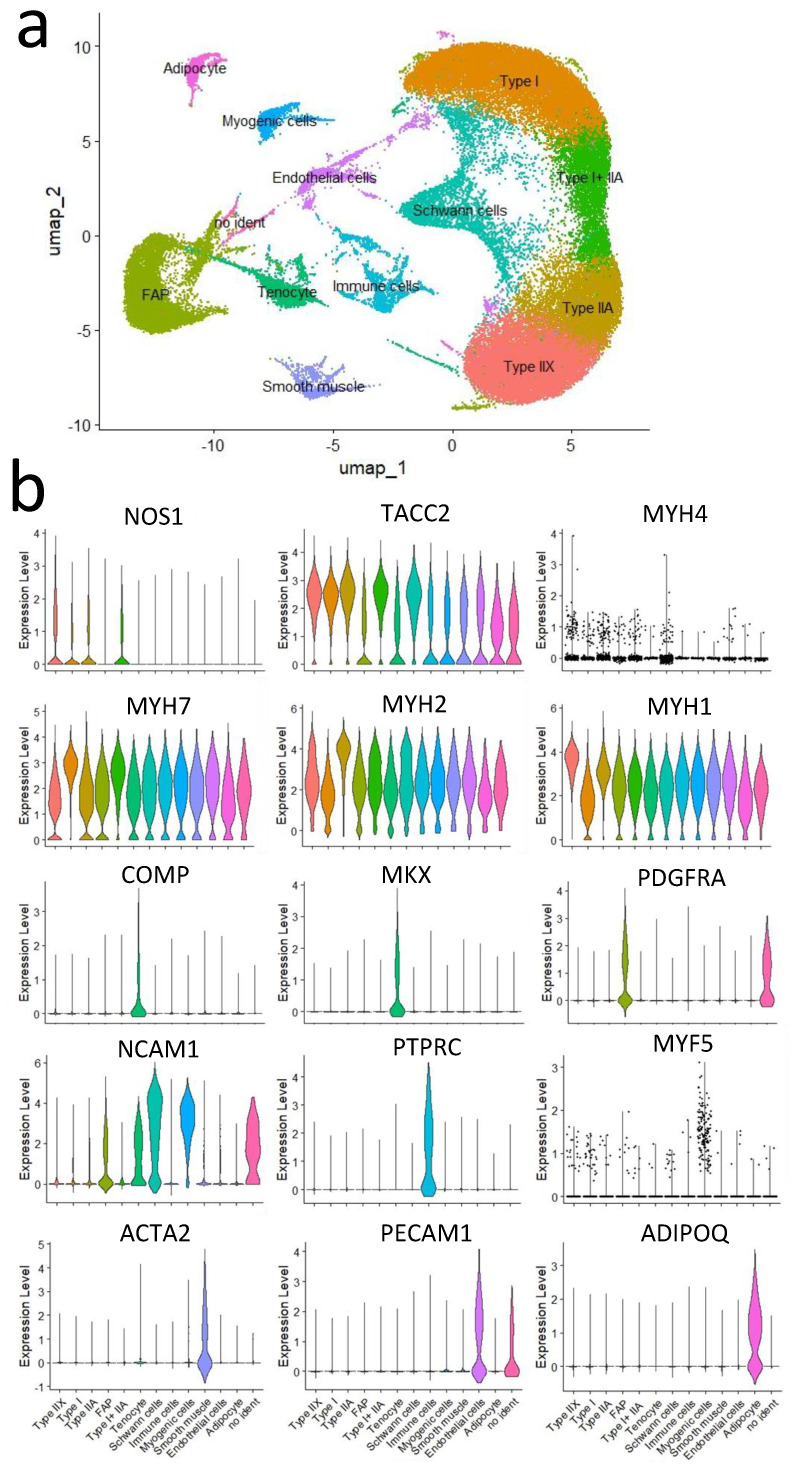
Cellular composition of the pectoralis and adductor muscles. (**a**) The uniform manifold approximation and projection (UMAP) plot illustrates the integrated data for the adductor muscle (Round) and pectoralis muscle (Brisket), classified into distinct clusters. Cell populations were classified into 13 clusters based on marker gene expression. (**b**) Violin plots of marker genes were used to identify cell populations in a dataset integrating Brisket and Round samples. The *y*-axis represents statistical significance, while the plots indicate the number of nuclei.

A clustering analysis using the Seurat R package classified these nuclei into 19 clusters. Subsequently, annotation analysis based on known marker genes identified 13 distinct cell populations (Figure 4a). For cell-type classification, the following marker genes were used (Table 3). Violin plots for each marker gene are shown (Figure 4b).

**Figure 5:**
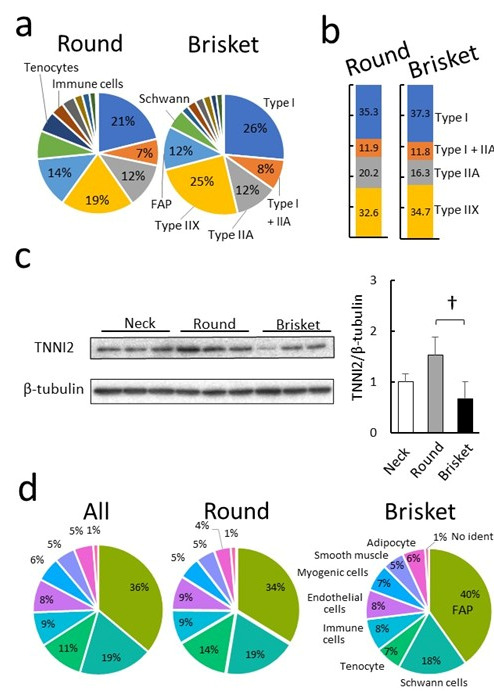
Proportions of cell populations. (**a**) The pie chart represents the distribution of cell populations, with numerical values indicating the percentage composition of the top five cell types. Myofibrillar types include Type I, Type IIA, and Type IIX fibers, along with fibro–adipogenic progenitors (FAPs). (**b**) Myofibrillar composition. The proportions of myofibrillar types (Type I, Type IIA, and Type IIX) are shown. (**c**) Quantification of protein expression by immunoblotting. TNNI2 is an isoform of troponin I expressed in fast-twitch Type IIA fibers. Tubulin was used as an internal control. Bar charts represent mean detection intensity. Error bars indicate ± SE. Significant differences are denoted as follows: † *p* < 0.05 (Tukey’s HSD test, *n* = 3). (**d**) Pie chart illustrating cell populations excluding myofiber.

The proportions of cell populations in Round and Brisket are shown using pie charts (Figure 5a). Among the detected nuclei, 60% originated from myofibers, representing the largest proportion. The second most abundant population was FAPs, followed by Schwann cells. A bar graph was used to compare myofiber cell populations between Round and Brisket (Figure 5b). Brisket samples contained approximately 2% more type I and IIX myofibers. Conversely, type IIA myofibers were 3.9% more abundant in Round than in Brisket samples. To examine protein expression levels in muscle tissue, a specific antibody targeting the fast-twitch muscle fiber protein TNNI2 was generated, and its expression was analyzed using Western blotting. TNNI2 expression was significantly higher in Round than in Brisket samples (Figure 5c).

Subsequently, we compared the proportions of non-myofiber cell populations. Compared to Round, the Brisket sample exhibited higher proportions of FAPs, myogenic cells, and adipocytes. In contrast, tendon cell proportions were higher in Round than in Brisket samples (Figure 5d).

**Figure 6:**
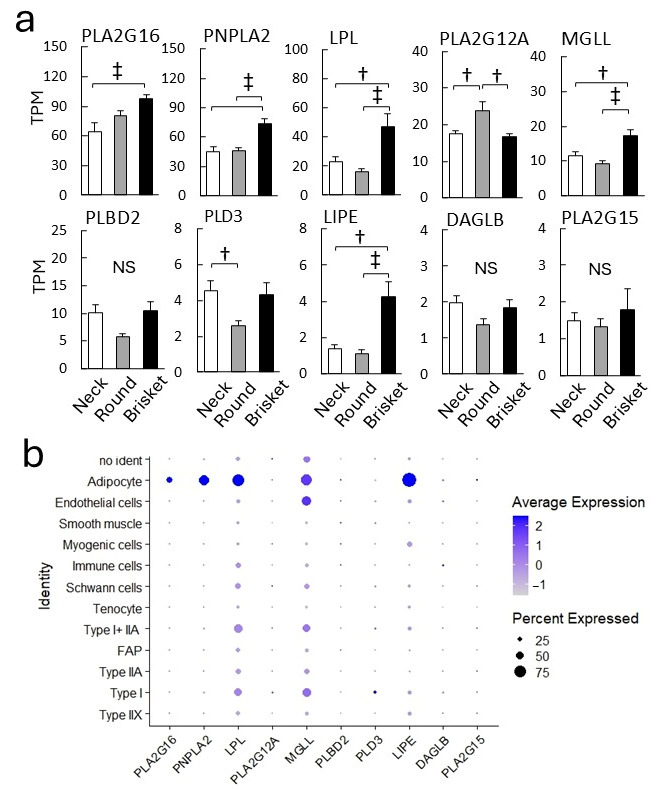
Cell populations expressing lipid-degrading enzymes. (**a**) Expression of lipase genes. The graph displays gene expression levels in the adductor muscle (Round), sternocleidomastoid muscle (Neck), and pectoral muscle (Brisket), shown as mean TPM (transcripts per million). Error bars indicate ± SE. Significant differences are denoted as follows: ‡ *p* < 0.01, † *p* < 0.05 (Tukey’s HSD test, *n* = 8), while nonsignificant differences are indicated as ns. (**b**) Dot plot showing lipase expression. The dot size represents the proportion of cells expressing the gene, while color intensity indicates statistical significance.

Japanese Black beef exhibits distinctive characteristics, including higher free fatty acid levels than other breeds [8]. However, its molecular mechanisms remain unclear. Triacylglycerol lipases and phospholipases are key enzymes in lipolysis; however, their expression patterns in adipocytes and muscle remain largely uncharacterized [35]. To address this, RNA-seq analysis identified ten lipases in muscle samples (Figure 6a). In Brisket samples, the expression levels of *phospholipase A2 group XVI* (*PLA2G16*), *patatin-like phospholipase domain-containing protein 2* (*PNPLA2*), *lipoprotein lipase* (*LPL*), *monoglyceride lipase* (*MGLL*), and *hormone-sensitive lipase* (*LIPE*) were significantly higher than in Neck and Round samples.

Subsequently, snRNA-seq was performed to analyze gene expression at the single-cell level (Figure 6b). *LIPE*, *LPL*, *PNPLA2*, *MGLL*, and *PLA2G16* were highly expressed in adipocytes. In contrast, *PLA2G12A* was highly expressed in myofibers. Further comparison of myofiber types revealed that *MGLL*, *LPL*, and *LIPE* were expressed at similar levels in Type I and Type I + IIA myofibers, whereas *phospholipase D3* (*PLD3*) and *PLA2G12A* were significantly upregulated in Type I myofibers.

**Figure 7:**
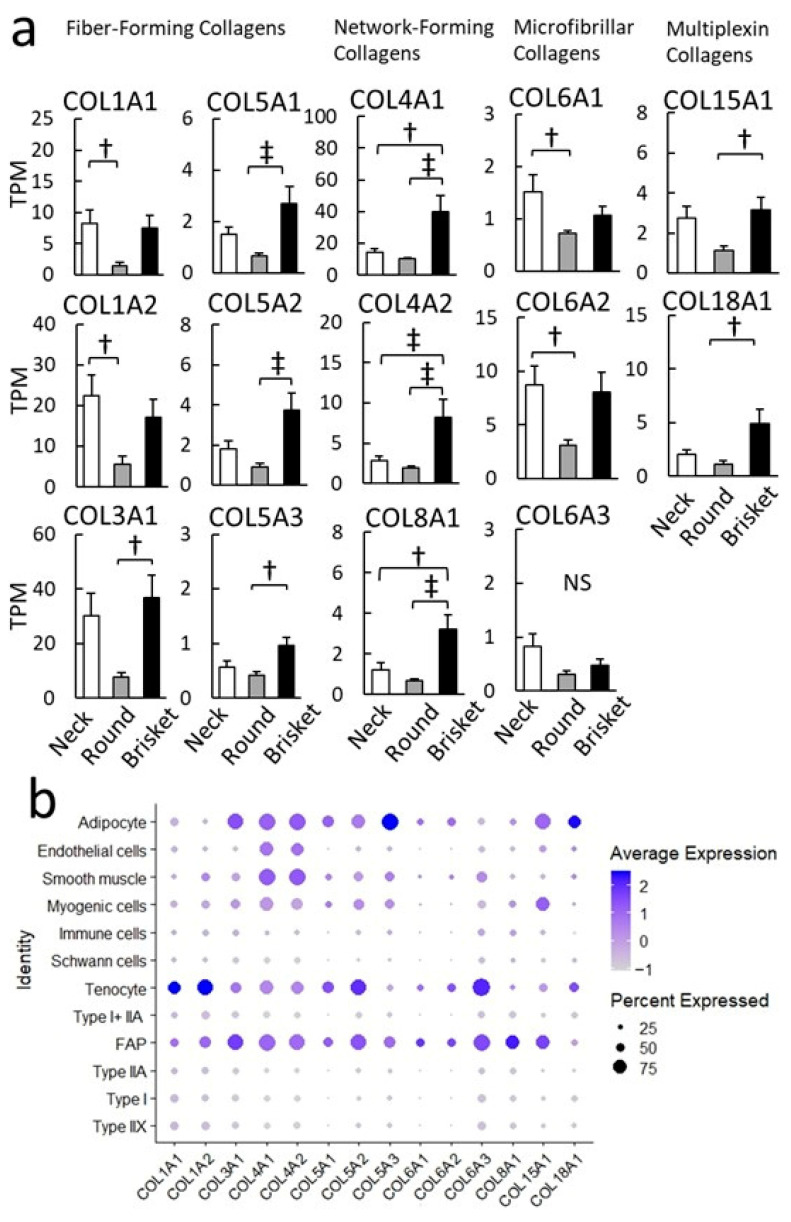
Cell populations expressing collagen isoforms. (**a**) Collagen expression. The graph displays gene expression levels in the adductor muscle (Round), sternocleidomastoid muscle (Neck), and pectoral muscle (Brisket), shown as mean TPM (transcripts per million). Error bars indicate ± SE. Significant differences are denoted as follows: ‡ *p* < 0.01, † *p* < 0.05 (Tukey’s HSD test, *n* = 8), while nonsignificant differences are indicated as ns. (**b**) Dot plot showing collagen expression. The dot size represents the proportion of cells expressing the gene, while color intensity indicates statistical significance.

Gene annotation linked Brisket samples to multiple ECM-related GO terms (Figure 2). To further explore ECM differences among muscle regions, we analyzed ECM component expressions. (Figure 7a). In Brisket samples, the expression levels of collagen IV (*COL4A1*, *COL4A2*) and collagen VIII (*COL8A1*), which form a sheet-like collagen network [36], were higher than in Neck and Round samples. Nonfibrillar multiplex collagens, including collagen XV (*COL15A1*) and collagen XVIII (*COL18A1*), also showed higher expression in Brisket samples.

In Neck samples, collagen type I (*COL1A1*, *COL1A2*) and collagen type VI (*COL6A1*, *COL6A2*) showed higher expression than Brisket and Round samples. The collagen gene expression profile was analyzed across different cell populations (Figure 7b). Major fibrillar collagens, such as *COL1A1* and *COL1A2*, were highly expressed in tenocytes. Another fibrillar collagen, *COL3A1*, was predominantly expressed in FAPs and adipocytes. The basement membrane, *COL4A1,* and *COL4A2*, were predominantly expressed in FAPs, smooth muscle cells, and adipocytes, with higher expression also in tenocytes, myogenic cells, and endothelial cells.

**Figure 8:**
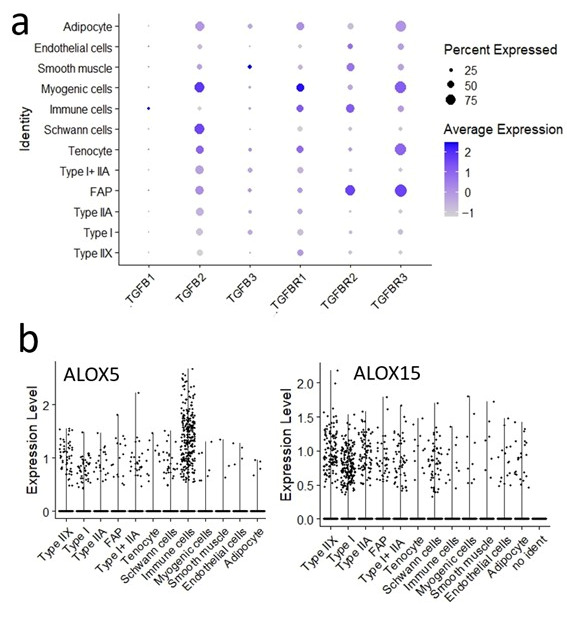
Cell populations expressing specific genes. (**a**) Dot plot showing the expression of transforming growth factor beta (TGF-β)-related genes. The dot size represents the proportion of cells expressing the gene, while color intensity indicates statistical significance. (**b**) Violin plots showing the distribution of arachidonic acid lipoxygenase (ALOX) isoforms in adductor muscle (Round) and pectoral muscle (Brisket) samples.

To examine crosstalk among ECM-associated cells, previously identified TGF-β-related genes [37] were analyzed (Figure 8a). TGF-β isoforms were highly expressed in myogenic cells and Schwann cells and detected in adipocytes, tenocytes, and myofibers. *TGFB1* was expressed in immune cells, whereas *TGFB3* was expressed in smooth muscle. Meanwhile, TGF-β receptors were highly expressed, with *TGFBR2* and *TGFBR3* in FAPs, *TGFBR3*, and *TGFBR1* in myogenic cells and tenocytes, and *TGFBR2* and *TGFBR1* in immune cells.

In the analysis of *arachidonic acid lipoxygenase* (*ALOX*) expression [38], *ALOX5* was found to be specifically expressed in immune cells, whereas *ALOX15* was expressed in myofibers, including Type I, Type IIX, and Type IIA myofibers (Figure 8b).

**Figure 9:**
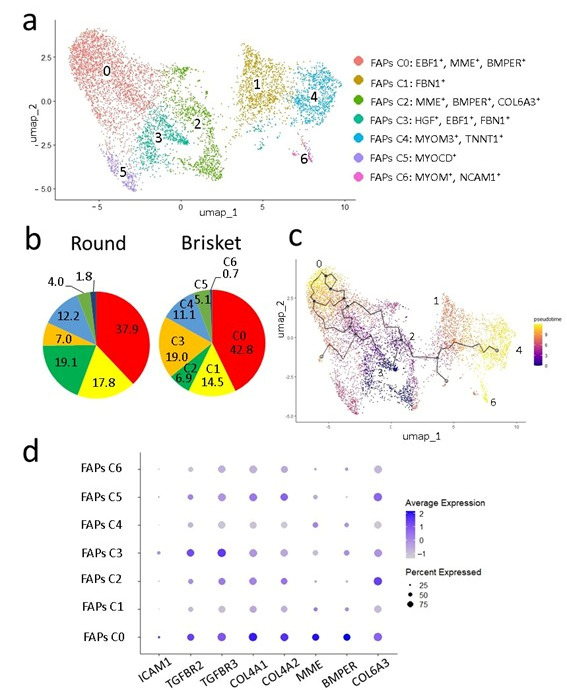
Pseudotime trajectory analysis of FAPs of Japanese Black cattle. (**a**) Clustering analysis of FAPs. FAPs were classified into seven clusters. Marker genes used to identify cell types are shown in the legend. (**b**) Pie chart showing the ratio of classified FAP clusters in pectoral muscle (Brisket) and adductor muscle (Round). (**c**) Pseudotime trajectory of FAPs at different developmental stages. The dot color indicates the degree of cell differentiation. Purple represents undifferentiated cells, while yellow represents differentiated cells. Arrows schematically indicate the direction of differentiation. (**d**) Dot plot showing the expression of marker genes related to the FAP cluster.

FAPs contribute to ectopic IMF accumulation in porcine skeletal muscle [39,40] and serve as IMF precursor cells in Japanese Black cattle [19]. To clarify the lineage of FAPs in IMF development in Japanese Black cattle, we further classified cell types within the FAP population (Figure 9a). Gene expression analysis identified seven FAP subtypes (C0–C6).

FAP clusters C0 and C2 expressed *EBF1* [39], *MME* [41], and *BMP-binding endothelial regulator* (*BMPER*) [42], indicating high adipogenic potential. In C0, *intercellular adhesion molecule-1* (*ICAM1*) [43] was expressed, whereas *thymocyte selection-associated high-mobility group box* (*TOX*) [44] was specifically expressed in C2. C1 and C3 expressed *fibrillin 1* (*FBN1*) [45], representing fibroblast-like FAPs [46], with C1 also expressing *COL6A1* [47]. C4 and C6 expressed the *myocyte marker myomesin-3* (*MYOM3*) [48], with C4 further expressing *TNNT1*, representing myofibroblast-like FAPs. C6, in contrast, expressed *neural cell adhesion molecule 1* (*NCAM1*/*CD56*) [44,49]. C5 expressed the pericyte marker *myocardin* (*MYOCD*) [50].

C0 was more abundant in Brisket than in Round samples (Figure 9b). C1 and C3, involved in fibrogenesis and myogenesis, were more abundant in Round than in Brisket samples. C2 and C3 were comparable between Brisket and Round samples. Trajectory analysis of clustered FAPs was analyzed using *Monocle3* (Figure 9c). The results suggest that C0, which has adipogenic potential, and C4 and C6, linked to myogenesis, were the most differentiated (Figure 9c). In contrast, C3, with the lowest differentiation state and high fibroblast marker expression, was the least differentiated, followed by C2, which expressed both adipogenic and fibroblast markers. A dot plot presents the expression levels of known [51,52] and newly identified marker genes in the FAP clusters (Figure 9d).

## 4. Discussion

### 4.1. Muscle Tissue Composition

In this study, NGS technology was used to characterize the cellular composition of muscle tissue in Japanese Black cattle and to identify genes associated with IMF. RNA-seq identified multiple genes associated with myofiber composition (Figure 5b). Neck and Brisket exhibited a high expression of *MYH7*, *TNNC1*, *TNNI1*, and *TNNT1*, characteristic of slow-twitch type I myofibers (Figure S5). In contrast, Round displayed a significantly higher expression of the fast-twitch type II genes *MYH1*, *TNNC2*, *TNNI2*, and *TNNT3*. The gene expression patterns of myosin-heavy chains in different muscle regions observed in this study align with previously reported findings in Japanese Black cattle [53].

### 4.2. Lipid Metabolism and Lipoxygenase

Brisket exhibited more IMF than Neck and Round (Figure 1b). Lipid-metabolism-related pathways were upregulated in Brisket (Figure 2), and key genes, including *SCD*, *FASN*, and *ELOVL6*, were more highly expressed in Brisket than in Neck and Round (Figure 3). These genes encode enzymes involved in the lipid biosynthesis pathway [52].

Several lipases, including *PLA2G16*, *PNPLA2*, *LPL*, *MGLL*, and *LIPE* (*hormone-sensitive lipase*), exhibited high expression in Brisket, originating from IMF (Figure 6). *PNPLA2* (*adipose triglyceride lipase*, *ATGL*) functions as a key lipase with *LIPE* and *MGLL* in triacylglycerol hydrolysis, generating free fatty acids in adipocytes [54]. *PNPLA2* has been linked to neutral lipid storage disease and IMF accumulation [55]. These lipases likely contribute to free fatty acid degradation during cold storage, known as wet aging [56].

The oxidation of lipid metabolites is crucial for flavor development, as it generates precursors to aromatic compounds [57]. In food, enzymatic oxidation is primarily driven by lipoxygenase-mediated oxidation of polyunsaturated fatty acids [58]. Research on lipoxygenase in meat is still in its early stages, and its precise role remains unclear [59]. In this study, among six ALOX isoforms [31], *ALOX5* was derived from the immune system, whereas *ALOX15* was found to be expressed in myofibers (Figure 8b). The activity of these enzymes may influence the generation of the sweet aroma of Japanese Black beef during cooking. However, the role of ALOX isoforms in lipid oxidation during postmortem aging remains unclear.

### 4.3. Collagen and Remodeling

In Brisket, ECM-related signal transduction pathways were upregulated (Figure 2). Several collagen isoforms were highly expressed in FAPs and adipocytes (Figure 7a) [60]. Various collagens, including *COL4As* secreted by FAPs and adipocytes, are considered as essential for microenvironment formation within muscle tissue (Figure 7). Collagen IV, a key component of the basement membrane surrounding IMF, comprises six isoforms that assemble into triple-helical structures in three distinct combinations [61,62]. snRNA-seq analysis suggested that *COL4A1* and *COL4A2* were broadly distributed among cell populations within muscle tissue while *COL4A3*, *COL4A4*, and *COL4A5* were predominantly expressed in Type I myofibers. Additionally, *COL4A5* and *COL4A6* were detected in Type IIA and Type IIX myofibers and near vascular smooth muscle (Figure S6).

In previous research [15], *COL4A5* and *COL4A6* were significantly upregulated in IMF compared to subcutaneous fat. *COL4A5* has been reported to promote the differentiation of adipose-derived stem cells through the YAP/TAZ pathway [63]. These findings suggest that *COL4As* contribute to ECM remodeling during IMF formation, potentially influencing adipocyte differentiation within the muscle microenvironment [64].

TGF-β signaling is known to induce collagen expression. Cell type classification of TGFB-related genes revealed a correlation between cell populations expressing *TGFBR2* and *TGFBR3* (Figure 8a) and those expressing collagen genes [65]. TGF-β plays a dual role in regulating fibrosis and adipogenesis; however, its precise function in intramuscular adipocyte differentiation remains unclear. Further investigation is required to determine whether TGF-β primarily promotes ECM deposition and fibrosis or facilitates adipogenesis within the IMF microenvironment.

### 4.4. Fibro–Adipogenic Progenitor Heterogeneity

FAPs play a critical role in regulating ECM and IMF deposition within muscle tissue [27,66]. In this study, the snRNA-seq analysis identified distinct FAP subtypes in Japanese Black cattle, suggesting heterogeneity in their functions and differentiation potential (Figure 9a). Notably, specific FAP clusters exhibited a high expression of adipogenic markers. Among these, *ICAM1* was specifically expressed in FAP C0 (Figure 9d), which exhibited high adipogenic potential and may be involved in preadipocyte differentiation and ECM remodeling [67]. Likewise, *TOX* was uniquely expressed in C2, displaying both adipogenic and fibroblastic characteristics. TOX belongs to a transcription factor family containing a highly conserved high-mobility group (HMG-Box) domain [68]. Since transcription factors are crucial for IMF differentiation [69,70], TOX may regulate the balance between these lineages. ICAM1 and TOX are novel markers that have not been previously identified in human disease models [26,27] or other livestock species [19,20,39]. In contrast, the *thyroid hormone-responsive* (*THRSP*) gene, which has been identified in porcine IMF [40], was not detected in Japanese Black cattle.

**Figure 10:**
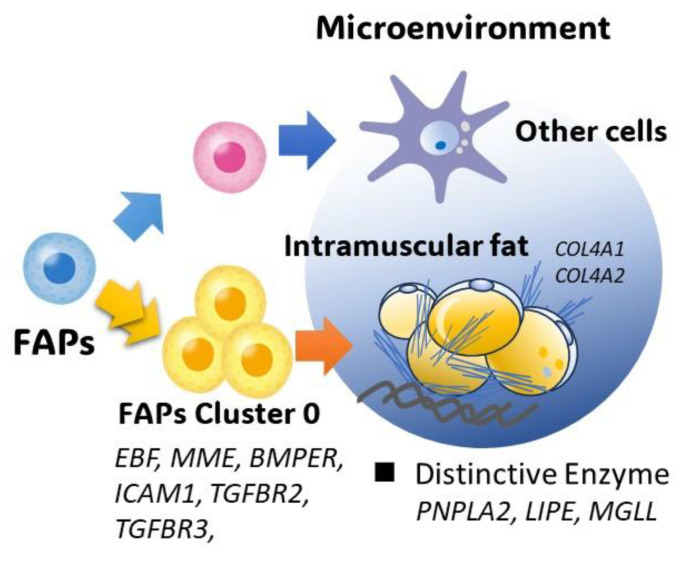
Relationship between intramuscular fat precursor cells and meat quality. The microenvironment surrounding intramuscular fat (IMF) in Japanese Black cattle is enriched with basement membrane components, including collagen (*COL4A1* and *COL4A2*), that are secreted by FAPs and adipocytes. In addition, adipocyte-specific lipases (e.g., *PNPLA2*, *LIPE*, and *MGLL*) are highly expressed in IMF. These unique tissue characteristics are thought to contribute to the distinct meat quality of Japanese Black beef. Furthermore, the muscle tissue of Japanese Black cattle harbors diverse fibro–adipogenic progenitors (FAPs) with varying adipogenic and fibrogenic potentials, which may influence differences in IMF deposition and marbling formation.

Furthermore, trajectory analysis revealed that a subset of FAPs in Brisket exhibited enhanced adipogenic potential compared to those in Round (Figure 9b). This suggests that regional differences in IMF accumulation may be influenced by the intrinsic properties of FAP subpopulations, as well as by localized ECM composition and signaling pathways Figure 10. Although FAPs have been extensively studied in other livestock species [34,71], their precise role in IMF formation in Japanese Black cattle remains incompletely understood.

Future studies should focus on elucidating the molecular mechanisms governing FAP differentiation and their interactions with other cell types, such as myogenic and adipogenic lineages. Additionally, investigating the epigenetic regulation and spatial transcriptomics of FAPs in Japanese Black cattle may provide further insights into the mechanisms underlying IMF deposition and its contribution to meat quality [72].

## 5. Conclusions

This study revealed differences in gene expression and FAP clusters associated with IMF accumulation among the muscle tissues of Japanese Black cattle. Furthermore, by analyzing lipase and lipoxygenase expression related to lipid metabolism, this study provides genetic insights into traits contributing to meat flavor influenced by IMF.

However, the genetic and epigenetic mechanisms underlying FAP heterogeneity remain unclear. Additionally, further investigation is required to elucidate the precise differentiation through which FAPs in Japanese Black cattle develop into intramuscular adipocytes. In this study, integrating multi-omics approaches, including epigenomic and spatial transcriptomics analyses, may help to clarify the regulatory networks controlling FAP differentiation and IMF deposition, addressing existing knowledge gaps in IMF formation.

A deeper understanding of these mechanisms will advance muscle science research and contribute to broader studies on ectopic fat deposition and metabolic regulation.

## Figures and Tables

**Table 1:** Summary of RNA-seq data for Japanese Black beef. The dataset includes sternocleidomastoid muscle (Neck), adductor muscle (Round), and pectoralis muscle (Brisket) results. The table presents the total read count used for mapping, the number of successfully mapped reads, and the mapping rate (% mapped reads), with mean values for each tissue (*n* = 8).

Muscle Tissue	Total Read (Count)	Mapped Read (Count)	Mapped Read (%)
Neck	70,838,677	70,609,753	99.7
Round	76,025,869	75,783,954	99.7
Brisket	70,529,128	70,263,281	99.6

**Table 2:** Summary of snRNA-seq for Japanese Black beef. The dataset includes pectoralis muscle (Brisket) and adductor muscle (Round) results.

Muscle Tissue	Estimated Number of Cells	Median Genes Per Cell	Reads Mapped to Genome (%)	Total Genes Detected
Brisket	38,939	1654	97.10%	25,580
Round	51,506	1573	96.80%	25,955

**Table 3:** Summary of marker genes. The markers were selected based on previously published papers [17,19,33,34].

Cell Type	Marker Gene
Myofibers	*Nitric oxide synthase 1* (*NOS1*)
	*Transforming acidic coiled-coil-containing protein 2* (*TACC2*)
	*Myosin heavy chain 1* (*MYH1*)
	*Myosin heavy chain 2* (*MYH2*)
	*Myosin heavy chain 7* (*MYH7*)
Fibro–adipogenic progenitors (FAPs)	*Platelet-derived growth factor receptor alpha* (*PDGFRA*)
Tenocytes	*Cartilage oligomeric matrix protein* (*COMP*)
	*Mohawk homeobox* (*MKX*)
Chwann cells	*Neural cell adhesion molecule 1* (*NCAM1*)
Immune cells	*Protein tyrosine phosphatase receptor type C* (*PTPRC*)
Myogenic cells (muscle satellite cells)	*Myogenic factor 5* (*MYF5*)
Smooth muscle cells	*Actin alpha 2* (*ACTA2*)
Endothelial cells	*Platelet and endothelial cell adhesion molecule 1* (*PECAM1*)
Adipocytes	*Adiponectin* (*ADIPOQ*)

## Data Availability

The data presented in this study are available on request from the corresponding author. The data are not publicly available due to patent issues.

## Supplementary Materials

The following supporting information can be downloaded at https://www.mdpi.com/article/10.3390/metabo15040231/s1, Figure S1: Quality testing of RNA. Figure S2: Quality assessment of RNA sequencing based on Phred quality score. Figure S3: Annotation analysis of the sternocleidomastoid muscle. Figure S4: Heatmap analysis of 52 upregulated DEGs in pectoral muscle. Figure S5: Expression of myofibrillar-related genes. Figure S6: Dot plot showing the expression of collagen type IV isoforms.

## Abbreviations

IMF	Intramuscular fat
scRNA-seq	Single-cell RNA sequencing
snRNA-seq	Single-nucleus RNA sequencing
NGS	Next-generation sequencing
RIN	RNA integrity
DEG	Differentially expressed gene
TPM	Transcripts per million
GO	Gene Ontology
KEGG	Kyoto Encyclopedia of Genes and Genomes
SE	Standard error
NS	Not significant
UMAP	Uniform manifold approximation and projection
