# Supplementary material for: Single-Nucleus RNA Sequencing Reveals Muscle-Region-Specific Differences in Fibro-Adipogenic Progenitors Driving Intramuscular Fat Accumulation
Source: Metabolites. 2025 Mar 28;15(4):231. doi: 10.3390/metabo15040231 (PMC12029141; doi:10.3390/metabo15040231)
Supplement: Supplementary file 1 [file metabolites-15-00231-s001.zip › metabolites-3543492-supplementary.pdf]

Muscle tissue	RNA concentration (ng/μl)	RIN
Neck	32.20 ± 6.53	6.71 ± 0.29
Round	35.17 ± 5.92	6.90 ± 0.17
Brisket	31.99 ± 6.46	6.94 ± 0.30

Figure S1: Quality testing of RNA.

The table presents results from the sternocleidomastoid muscle (Neck), adductor muscle (Round), and pectoralis muscle (Brisket) results. RNA concentration and RNA integrity number (RIN) are presented as mean ± standard deviation for each tissue (n = 8).

Muscle tissue	Number of leads	Number of bases in nucleic acids	Q30R1 (%)	Q30R2 (%)
Neck	70,838,677	10,625,801,513	92.2	92.3
Round	76,025,869	11,403,880,388	92.2	92.0
Brisket	70,529,128	10,579,369,238	91.9	92.1

Figure S2. Quality assessment of RNA sequencing based on Phred Quality Score.

The table includes sternocleidomastoid muscle (Neck), adductor muscle (Round), and pectoralis muscle (Brisket) results. Q30 was calculated based on the Phred Quality Score (mean value for each tissue, n = 8).

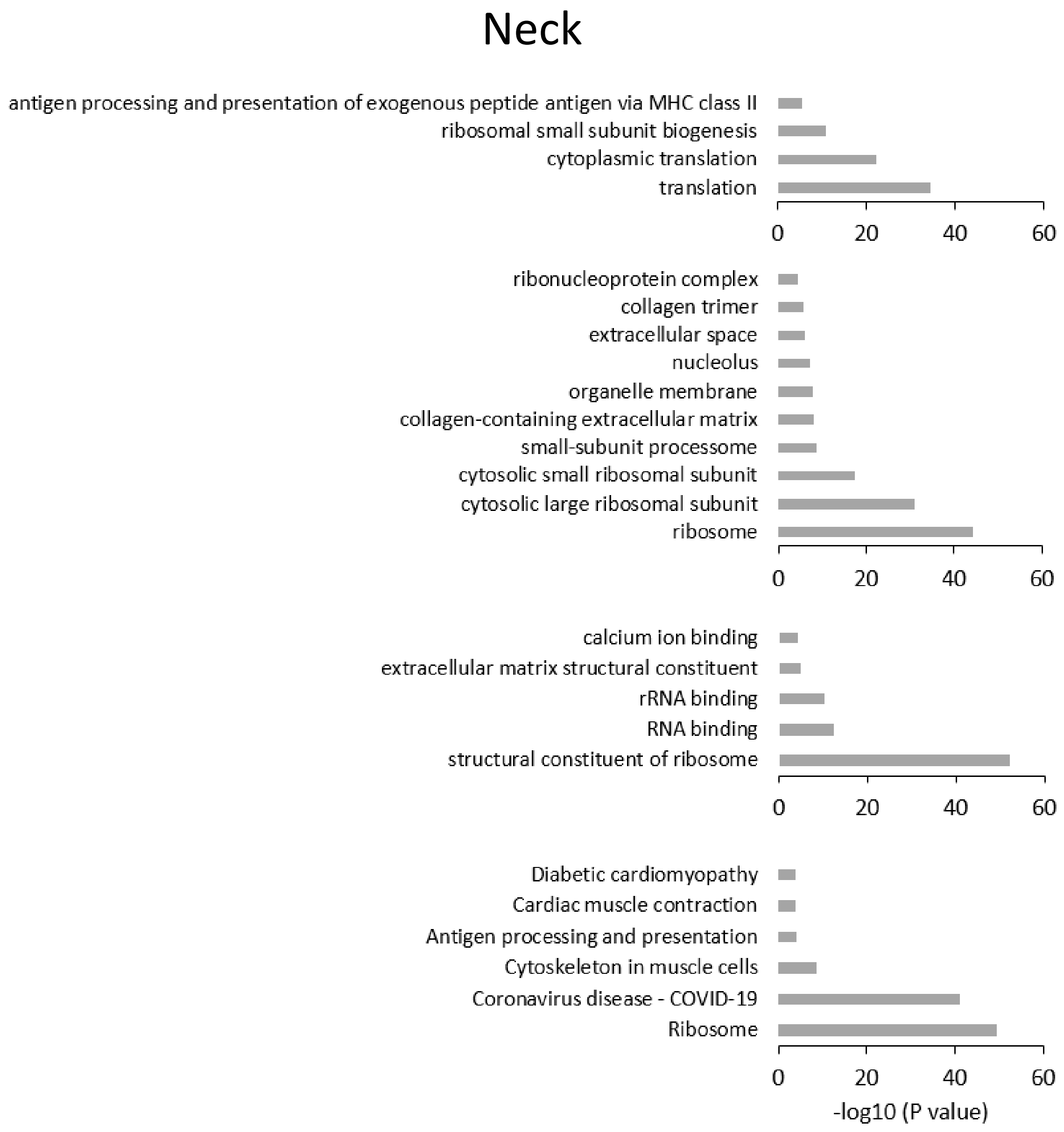

Figure S3. Annotation analysis of the sternocleidomastoid muscle. (a) Gene Ontology (GO) analysis and KEGG pathway analysis were conducted using DAVID for 621 upregulated genes in the sternocleidomastoid muscle (Neck).

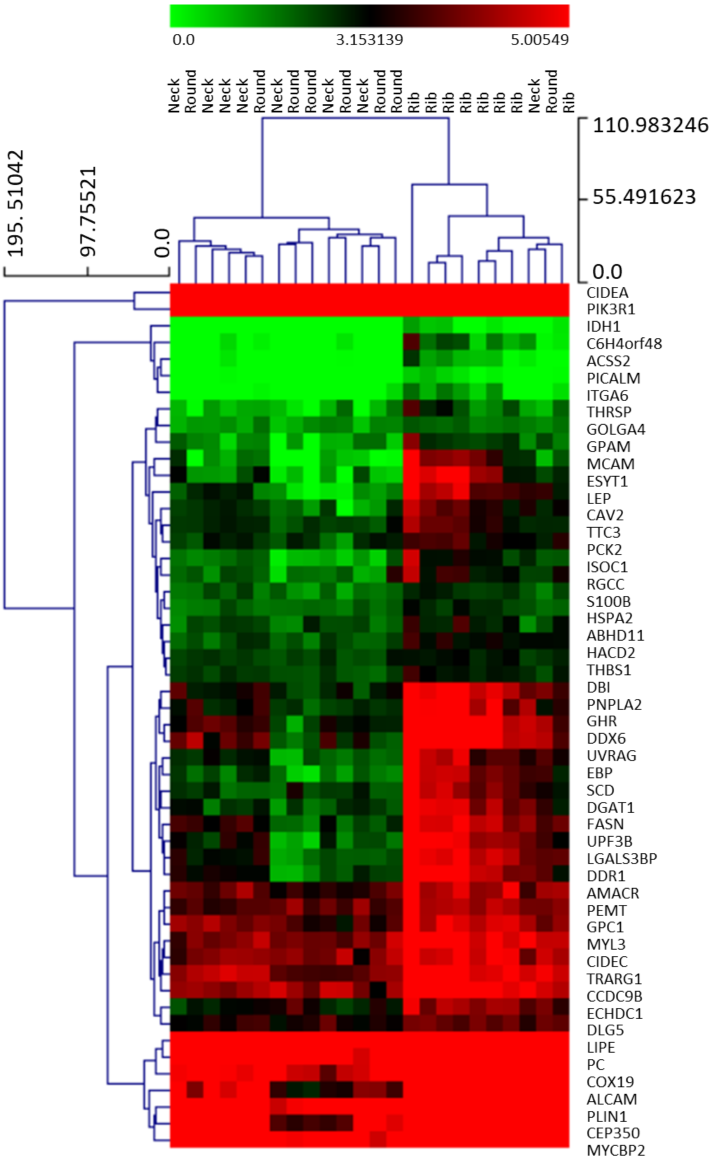

Figure S4. Heatmap analysis of 52 upregulated DEGs in pectoral muscle. The heatmap compares the pectoral muscle (Brisket), adductor muscle (Round), and sternocleidomastoid muscle (Neck). Red indicates high expression, while green indicates low expression.

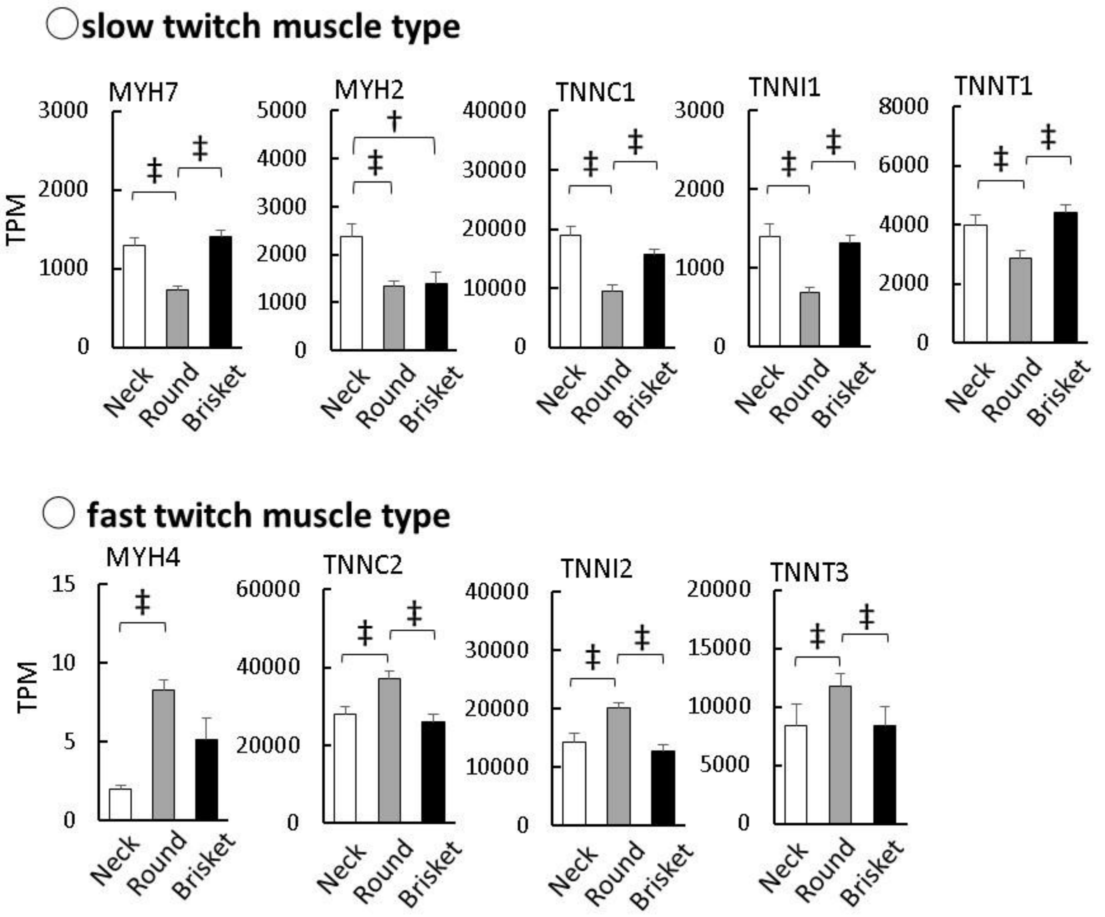

Figure S5. Expression of myofibrillar-related genes. The graph displays gene expression levels in the adductor muscle (Round), the sternocleidomastoid muscle (Neck), and the pectoral muscle (Brisket), represented as the mean TPM (Transcripts Per Million). Error bars indicate ± SE. Significant differences are denoted as follows: ‡$p < 0.01$, †$p < 0.05$ (Tukey's test, n = 8).

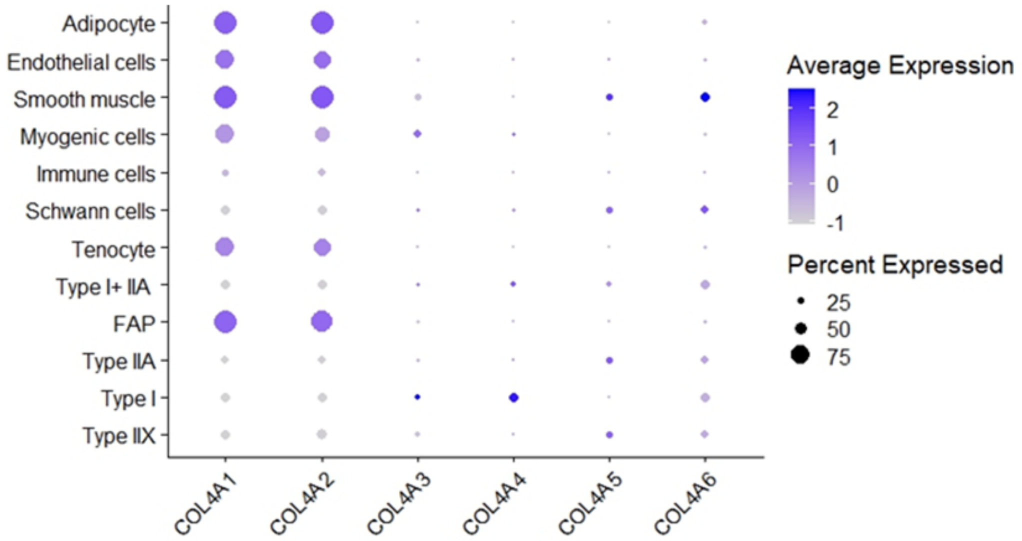

Figure S6. Dot plot showing the expression of collagen type IV A isoforms.
The dot size represents the proportion of cells expressing the gene, while the color intensity reflects the expression level.
